# Association between physical activity performed at different intensities and cardiovascular health in patients with peripheral artery disease: an observational study

**DOI:** 10.31744/einstein_journal/2024AO0682

**Published:** 2024-09-25

**Authors:** Max Duarte de Oliveira, Wellington Segheto, Hélcio Kanegusuku, Aline Mendes Gerage, Nelson Wolosker, Marilia de Almeida Correia, Raphael Mendes Ritti-Dias

**Affiliations:** 1 Universidade Nove de Julho São Paulo SP Brazil Universidade Nove de Julho, São Paulo, SP, Brazil.; 2 Hospital Israelita Albert Einstein São Paulo SP Brazil Hospital Israelita Albert Einstein, São Paulo, SP, Brazil.; 3 Universidade Federal de Santa Catarina Florianópolis SC Brazil Universidade Federal de Santa Catarina, Florianópolis, SC, Brazil.; 4 Universidade de São Paulo Faculdade de Medicina Hospital das Clínicas São Paulo SP Brazil Hospital das Clínicas, Faculdade de Medicina, Universidade de São Paulo, São Paulo, SP, Brazil.

**Keywords:** Peripheral arterial disease, Intermittent claudication, Sedentary behavior, Accelerometry, Cardiovascular diseases

## Abstract

**Objective:**

To analyze the association between the time spent in sedentary behavior and physical activity of different intensities with cardiovascular health in men and women with peripheral artery disease.

**Methods:**

Two hundred and sixty patients with peripheral artery disease and claudication symptoms (65.7% men; 66±1 years; ankle brachial index 0.57±0.18) were evaluated. Physical activity and sedentary behavior were assessed using an accelerometer. Physical activity was classified into light and moderate-vigorous intensities. The cardiovascular outcomes included blood pressure (oscillometric method), cardiac autonomic modulation (heart rate variability), and arterial stiffness (pulse wave velocity).

**Results:**

Women spent more time engaged in light physical activity than men (341±14 min/day *versus* 306±9 min/day; p=0.040, respectively). There was no significant difference in the time spent on sedentary behavior and moderate-vigorous physical activity. Women had a higher systolic blood pressure (p=0.025), higher augmentation index (p<0.001), and lower sympathovagal balance (p=0.047) than men. Pulse wave velocity was only negatively associated with light physical activity (β= -4.66; 95%CI= -8.57; -0.76) in men. Light and moderate-vigorous physical activity and sedentary behavior were not associated with other cardiovascular outcomes.

**Conclusion:**

Higher levels of light physical activity were associated with lower arterial stiffness in men with peripheral artery disease.

## In Brief

We examined the sedentary behavior and physical activity of 260 patients with peripheral artery disease. Women engaged in more light physical activity than men did. Light physical activity was associated with lower arterial stiffness in men only, while no significant associations were found between sedentary behavior, moderate-vigorous physical activity, and cardiovascular outcomes.

## Highlights

◼ Women with peripheral artery disease exhibited higher blood pressure and arterial stiffness than men.
◼ Low levels of physical activity, particularly moderate to vigorous activity, were observed in individuals with peripheral artery disease.

## Graphical abstract

**Figure:**
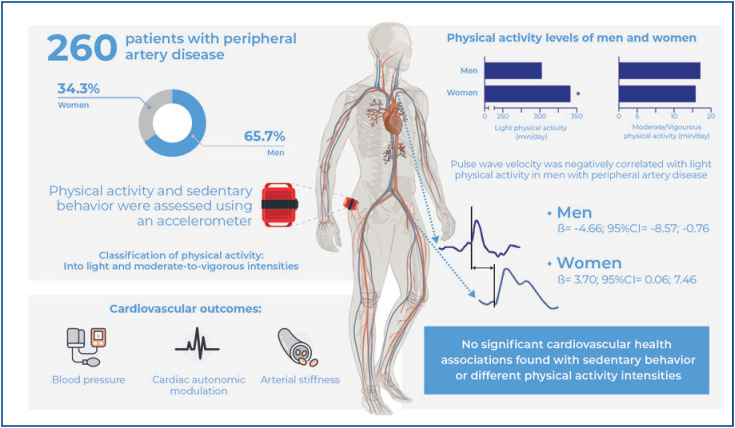

## INTRODUCTION

Peripheral artery disease (PAD) is a chronic disease caused by atherosclerosis that compromises blood irrigation in the extremities of the body, with the lower limbs being the most affected.^(1)^ Peripheral artery disease affects more than 202 million people worldwide and approximately 10.5% of Brazilians.^(2, 3)^ Patients with PAD and claudication symptoms have been shown to present with several alterations in cardiovascular function, including increases in blood pressure^(2)^ and arterial stiffness^(4,5)^ and cardiac autonomic control impairments^(5)^ which seem to be worse in women.^(6)^ In this context, it has been observed that women with PAD experience a more substantial disease burden than men, regardless of age disease severity and duration of the disease.^(7)^

The daily lives of patients with PAD and intermittent claudication are mainly composed of sedentary behaviors and physical activities (PA) performed at light intensity^(8,9)^ with only 3.4% achieving the recommended amount of moderate-vigorous PA.^(8)^ Notably, previous research has indicated that women report higher amounts of light PA and lower amounts of moderate-vigorous PA than men.^(10)^ In patients with PAD, total daily PA levels have been associated with better functional capacity,^(11)^ cardiovascular^(12)^ and cognitive functions^(13)^ while the time spent in sedentary behavior is related to higher arterial stiffness parameters^(5,14)^ and worse levels of cardiometabolic biomarkers.^(15)^ Considering that few patients practice moderate-vigorous PA, performing mostly light PA, and that there is a difference in PA partners between the sexes, this study investigated the association between light PA and cardiovascular health parameters in women and men with peripheral artery disease.

The hypothesis of this study was that more time spent doing light PA is associated with better cardiovascular health.

## OBJECTIVE

To analyze the association between sedentary behavior, physical activity of different intensities, and cardiovascular health in men and women with peripheral arterial disease.

## METHODS

### Participants

This was an observational cross-sectional study involving patients with PAD. Data were collected from 2015 to 2019. This study was approved by the research ethics committees of *Hospital Israelita Albert Einstein* (CAAE: 42379015.3.0000.0071; #4.435.316) and *Hospital das Clínicas da Faculdade de Medicina da Universidade de São Paulo* (CAAE: 42379015.3.3002.0068; # 4.614.795) and only started after its approval. All the patients signed an informed consent form to participate in the study. Inclusion criteria for the study were: I) ≥50 years of age, II) ankle-brachial index <0.90 in one or both legs, and III) presenting symptoms of intermittent claudication. Patients for whom it was not possible to measure the ankle-brachial index and those who presented with comorbidities limiting PA were not included in this study. The sample size was estimated with the objective of adjusting the linear regression models, with PA intensity as an explanatory variable and cardiovascular health indicators as outcomes, separately. As we did not observe a similar study in the literature, we arbitrarily anticipated the correlation between PA levels and the outcome at 0.28 with alpha = 0.05 and a power of 80%, the resulting estimated sample required for sufficient statistical power was 111 patients.

Recruitment was performed at two hospitals in São Paulo city. At the first visit, screening was performed to identify the general and clinical characteristics of the patients, information regarding their lifestyle, and ankle-brachial index values. Additionally, patients were equipped with accelerometers to assess the level of habitual PA and sedentary behavior. After approximately one week, the patients returned their accelerometer, and anthropometric and cardiovascular health parameters were assessed.

### Sample characteristics

Sex, age, education, marital status, skin color, and occupation were assessed through interviews. Comorbid conditions were identified according to medical records. Current smoking was defined as the consumption of cigarettes, cigars, and pipes in the previous six months. Body weight and height were measured using an electronic scale, and body mass index was calculated. The waist-hip ratio was calculated by measuring the waist and hip circumference in centimeters. Obesity was defined as a body mass index ≥30kg/m^2^ or waist-hip ratio ≥1.03 for men and 0.90 for women. The severity of PAD was determined using the ankle-brachial index, as previously described.^(13)^

### Physical activity level

Physical activity and sedentary behavior were assessed using accelerometers (ActiGraph, model GT3X+, USA). Each patient was instructed to use the accelerometer for seven consecutive days, removing it only when sleeping, showering, or performing water activities.^(16)^ The equipment was attached to an elastic belt and fixed on the right side of the hip. For analysis purposes, a minimum of 10 hours of daily activity recordings for at least four days, three weekdays and one weekend day, were considered valid data. To classify the level of habitual PA, cutoff points were calculated for the average minutes spent in each intensity of PA, based on a previous study by Freedson et al,^(17)^ classifying activities as sedentary behavior (0-99 counts/min), light (100-1951 counts/min), and moderate/vigorous (≥1952 counts/min) PA, adjusting for the time and number of days the device was worn.

### Cardiovascular outcomes

Brachial blood pressure was assessed using a monitor (HEM-742, Omron Healthcare, OsakaJapan). For this measurement, the participants remained in a sitting position for 10 min. Three consecutive measurements were performed, one minute apart, on both arms, with a cuff size adequate for arm circumference. The value used was the average of the last two measurements, as recommended by the Brazilian Guidelines of Hypertension.^(18)^

The autonomic modulation of the cardiovascular system was evaluated using the heart rate variability technique. Therefore, the patients remained in supine position for 10 min, during which the R-R intervals were recorded (the R-R interval is the distance between two successive R waves), using a heart rate monitor valid for this function (Polar RS800CX, Polar® Electro, Finland). A signal with at least 5 min of stationary signal was considered valid.

The R-R intervals were exported to the Kubios HRV program (Biosignal Analysis and Medical Imaging Group, Kempele Finland), and analyses were performed in time and frequency domains. Time-domain parameters, the standard deviation of all R-R intervals, and the root mean square of the differences between adjacent normal R-R intervals were obtained.^(19)^The frequency domain parameters were obtained via spectral analysis using the autoregressive method. Frequencies between 0.04 and 0.40 Hz were considered physiologically significant, with the low frequency (LF) and high frequency (HF) components represented by the oscillations between 0.04 and 0.15 Hz and 0.15 and 0.40 Hz, respectively. The power of each spectral component was calculated in normalized terms by dividing the power of each band by the total power, from which the very low frequency band value (<0.04 Hz) was subtracted, and the result was multiplied by 100.^(19)^

Arterial stiffness was estimated by evaluating the carotid-femoral pulse wave velocity. Applanation tonometry was used for this purpose. The carotid-femoral pulse waves were recorded sequentially by transcutaneous transducers, positioned above the right carotid and femoral arteries, using an applanation tonometry device (SphygmoCor^®^, AtCor Medical, Sydney, Australia).

Electrocardiographic recordings were obtained simultaneously with the carotid-femoral aortic pulse wave measurements as a reference standard to calculate the wave transit time. Two surface distances were measured by the investigator: one between the recording point on the carotid artery and sternal notch (distance 1) and the other between the sternal notch and recording point on the femoral artery (distance 2). The distance traveled by the pulse wave was calculated as "distance 2" - "distance 1." The carotid-femoral aortic pulse wave was calculated as: carotid-femoral aortic pulse wave = distance traveled by the pulse wave ¼ (m) / transit time(s).^(4,20)^ Augmentation Index was estimated in the radial artery, using the validated transfer function provided by the SphygmoCor^®^ software.^(21)^

### Statistical analysis

Data analysis and storage was performed using the statistical program SPSS (SPSS^®^, version 25.0, SPSS Inc, Chicago, IL). Data normality was verified using the Kolmogorov-Smirnov test, and Levene's test was used to analyze the homogeneity of variances. Continuous variables were summarized and presented as mean and standard deviation or as median and interquartile range, while categorical variables were summarized and presented as relative frequency.

Characteristics were compared between the sexes using the Student's *t*-test for independent samples and the χ^2^ test for categorical variables. The association between time spent in sedentary behavior and in mild and moderate-vigorous PA with cardiovascular outcomes was assessed using multiple linear regression. The analysis was extracted by sex, and a crude analysis and an adjusted model (age, ankle-brachial and waist circumference) were used as possible confounders. Multicollinearity analysis was performed assuming variance inflation factors smaller than five and tolerance below 0.20. For all analyses, significance was accepted at p<0.05.

## RESULTS

The characteristics of the participants and a comparison of cardiovascular parameters between men and women are shown in table 1. The sample comprised 260 participants, most of whom were male (65.7%). General characteristics were similar between sexes (p>0.050), except for body mass index (p=0.021) and time spent in light PA (p=0.040), which were higher in women than in men. Women had higher systolic blood pressure (p=0.025) and augmentation index of arterial stiffness (p<0.001) than men. The LF/HF was higher in men than in women (p=0.047).

**Table 1:** General characteristics and cardiovascular parameters of participants with peripheral artery disease

		Men n=171	Women n=89	p value
Age, years†		66±1	66±1	0.799
Body mass Index, kg/m²†		26.8±0.3	28.4±0.6	0.021
Ankle brachial Index†		0.56±0.01	0.59±0.02	0.232
Risk factors and comorbid conditions, % yes				
	Heart failure§	11.6	8.9	0.526
	Coronary artery disease§	31.5	30.3	0.495
	Diabetes§	61.6	38.4	0.097
	Dyslipidemia§	71.9	75.2	0.162
	Hypertension§	77.5	76.6	0.734
	Obesity§	18.1	39.3	0.217
	Renal failure§	14.0	16.8	0.510
	Smoker§	22.8	16.8	0.247
Medication, % yes				
	Angiotensin converting enzyme inhibitors§	30.7	26.1	0.496
	Angiotensin II receptor antagonists§	15.2	29.2	0.015
	Beta blockers§	29.8	37	0.365
	Calcium blocker§	17.5	26.9	0.095
	Diuretics§	30.4	29.2	0.756
	Peripheral vasodilator§	31.5	26.1	0.428
	Platelet antiaggregant§	66.6	66.2	0.377
	Statin§	30.7	85.5	0.387
Time in activities				
	Sedentary, min/day†	637±9	603±16	0.081
	Light physical activities, min/day†	306±9	341±14	0.040
	Moderate/vigorous physical activities, min/day†	17±1	16±4	0.563
Blood pressure				
	Systolic, mmHg‡	135.0 (133.3-139.6)	142.9 (138.3-148.6)	0.025
	Diastolic, mmHg‡	73.5 (72.1-75.3)	72.0 (71.1-76.5)	0.805
Arterial stiffness				
	Augmentation Index, %‡	26.4 (24.6-27.9)	31.6 (29.3-33.4)	<0.001
	Pulse wave velocity, m/s‡	9.0 (9.1-9.9)	9.0 (8.1-9.6)	0.492
Heart rate variability				
	SDNN, ms‡	34.2 (4.1-58.3)	30.4 (34.8-58.4)	0.326
	RMSSD, ms‡	28.2 (38.9-60.1)	23.0 (33.0-62.2)	0.808
	Low frequency/High frequency‡	1.1 (1.7-2.5)	0.9 (1.0-1.8)	0.047

† Data are presented as mean±Standard deviation

§ Data are presented as relative frequency

‡ Data are presented as median (interquartile range).

RMSSD: root mean square of differences between adjacent normal RRs; SDNN: standard deviation of all intervals.

Table 2 shows the results of the regression analysis for sedentary behavior, PA, and cardiovascular outcomes in men and women. Pulse wave velocity was negatively associated with light PA (β= -4.56; 95%CI= -8.46; 0.65) in men.

**Table 2:** Crude and adjusted analysis of the association between time spent in sedentary behavior and different intensities of physical activities and cardiovascular outcomes in patients with peripheral artery disease

	Sedentary		Light physical activity		Moderate-vigorous physical activity	
	Crude	Adjusted*	Crude	Adjusted*	Crude	Adjusted*
	β (95%CI)	β (95%CI)	β (95%CI)	β (95%CI)	β (95%CI)	β (95%CI)
Men (n=171)						
SBP, mmHg	3.70 (-25.93; 33.35)	6.35 (-22.60; 35.32)	-6.93 (-29.88; 16.00)	0.59 (-23.97; 22.78)	0.79 (-6.49; 6.33)	0.14 (-6.31; 6.60)
DBP, mmHg	-2.16 (-16.83; 12.51)	0.89 (-15.36; 13.57)	10.48 (-0.73; 21.69)	7.85 (-4.00; 19.17	2.06 (-1.11; 5.24)	2.48 (-0.62; 5.58)
AI, %	-1.99 (-17.10; 13.11)	0.29 (-14.93; 14.33)	3.89 (-7.70; 15.49)	1.37 (-10.34; 13.09)	0.71 (-3.96; 2.53)	0.45 (-3.69; 2.78)
PWV, m/s	1.40 (-21.43; 9.42)	0.96 (-4.11; 6.04)	-6.13 (-9.95; -2.30)	-4.56 (-8.46; 0.65)	0.33 (0.80; 1.46)	0.28 (0.85; 1.42)
SDNN, ms	14.83 (-53.93; 83.61)	9.57 (-60.19; 79.34)	-32.95 (-86.02; 20.12)	-27.75 (-83.89; 38.34)	-2.25 (-17.82; 13.31)	-1.18 (-16.75; 14.38)
RMSSD, ms	8.24 (-87.10; 103.57)	0.28 (-59.50; 84.98)	-55.89 (-129.14; 17.35)	-39.62 (-116.63; 37.39)	-6.36 (-27.71; 14.97)	-5.36 (-26.82; 16.08)
LF/HF	0.81 (-2.78; 4.40)	1.34 (-2.13; 4.86)	2.56 (0.91; 5.31)	1.81 (-1.00; 4.62)	0.28 (0.48; 1.06)	0.26 (0.52; 1.04)
Women (n=89)						
SBP, mmHg	-40.38 (-106.83; 26.06)	-36.74 (-103.11; 29.63)	-5.77 (-32.7; 21.9)	0.04 (-28.08; 28.17)	0.38 (-11.49; 12.26)	1.30 (-10.98; 13.60)
DBP, mmHg	10.47 (-19.53; 40.47)	-8.16 (-32.37; 38.69)	10.59 (-1.26; 22.44)	8.56 (-4.14; 21.22)	1.87 (-3.43; 7.17)	1.84 (-3.74; 7.45)
AI, %	2.36 (-24.26; 28.99)	-1.10 (-25.43; 27.63)	10.35 (-0.09; 20.79)	9.01 (-1.94; 19.96)	2.26 (-2.41; 6.95)	3.00 (-1.81; 7.82)
PWV, m/s	-4.44 (-13.72; 4.82)	-4.52 (-13.93; 4.87)	2.93 (-0.67; 6.54)	3.70 (0.06; 7.46)	0.42 (-1.25; 2.10)	0.23 (-1.54; 2.00)
SDNN, ms	172.25 (23.51; 320.99)	182.76 (-1.42; 237.56)	23.02 (-37.31; 83.36)	31.72 (-29.95; 93.39)	-11.63 (-39.21; 15.94)	-8.71 (-37.01; 19.58)
RMSSD, ms	206.85 (22.22; 391.49)	231.02 (-1.62; 407.84)	1.53 (-73.50; 76.57)	18.76 (-57.29; 84.82)	-13.33 (-47.50; 20.83)	-7.45 (-42.18; 27.24)
LF/HF	2.54 (-2.43; 7.51)	1.84 (-3.06; 6.75)	1.51 (0.39; 3.42)	1.11 (0.86; 3.09)	0.43 (0.42; 1.34)	0.29 (0.61; 1.20)

* Adjusted for age, ankle-brachial index, and waist circumference.

ß (95%CI): Regression coefficient (95% confidence interval); SBP: systolic blood pressure; DBP: diastolic blood pressure; AI: augmentation index; PWV: pulse wave velocity; RMSSD: root mean square of differences between adjacent normal RRs; SDNN: standard deviation of all intervals; LF: low frequency; HF: high frequency.

## DISCUSSION

The results of this study indicated that (i) women had higher systolic blood pressure, higher augmentation index, and lower sympathovagal balance than men; (ii) women spent more time in light PA than men, but there was no significant difference in time spent in sedentary behavior and moderate or vigorous PA; (iii) longer time spent in light PA was associated with lower pulse wave velocity in men; and (iv) time spent in sedentary behavior and moderate/vigorous PA were not associated with any indicator of cardiovascular health in men and women.

In this study, statistical analyses were performed according to gender because of the differences in functional and cardiovascular parameters between men and women with PAD.^(22)^ In line with previous literature, in this study, women with PAD had higher systolic blood pressure and augmentation index (an indicator of arterial stiffness) values than men with PAD.^(23)^However, men presented higher values of cardiac sympathetic modulation than women, which contrasts with the findings of Correia et al.,(23) who observed similar cardiac autonomic modulation between men and women with PAD.^(23)^ The differences in the prevalence of men taking medications that influence cardiac autonomic regulation, such as beta-blockers, are a potential cause of this controversy.

In this study, a disparity in the time devoted to light PA between men and women was observed, indicating that male participants dedicated less time to this form of PA than their female counterparts, which aligns with previous findings in similar studies.^(24)^

The association between time spent in light PA and pulse wave velocity, a marker of arterial stiffness, was evident only in men. Although this result is in line with earlier studies that observed an association between light PA and arterial stiffness in other populations^(25)^ the occurrence of this association in men is intriguing. The fact that men spent less time in light PA than women could highlight a potential threshold effect, in which a certain duration or intensity of light PA becomes more influential on arterial stiffness in men. Additionally, hormonal differences between men and women could contribute to this gender-specific association because hormonal fluctuations have been linked to vascular health. Furthermore, considering that men and women may engage in different types of light PA, the specific nature and intensity of these activities may vary and affect arterial stiffness differently based on gender. Further research is needed to elucidate the potential role of sex in the relationship between PA practices and arterial stiffness in patients with PAD.

Sedentary behavior was not associated with cardiovascular outcomes in men or women with PAD. Previously, Farah et al.^(15)^ observed associations between longer time spent in sedentary behavior and poor cardiometabolic biomarker levels (*i.e*., C-reactive protein, fibrinogen, plasminogen activator inhibitor 1 activity, and high-density lipoprotein cholesterol) in patients with PAD with claudication symptoms. Collectively, these findings indicate that sedentary behavior may exert a more pronounced influence on cardiometabolic biomarkers than clinical variables such as blood pressure, cardiac autonomic modulation, and arterial stiffness in this population. However, the observational nature of our study restricted to establish causality. Future studies with larger and more diverse samples are required to confirm these findings and explore potential causal relationships.

Time spent in moderate-vigorous PA was not associated with cardiovascular parameters in either men or women. In this study, the average time spent in moderate-vigorous PA was 16 min/week for men and women, with only 12.5% of men and 5.2% of women reaching the minimum recommendation for PA for the elderly (150 min/week of moderate-vigorous PA). Finally, although the ß coefficients in women were almost the half of observed in men, we cannot discard that the small number of women who met the recommendations might have affect the statistical power of the analysis, limiting the chance to find significant assocations in women.

The cross-sectional design of the study should be highlighted as a limitation, because no causality can be inferred. Therefore, longitudinal studies are required to investigate the mechanisms underlying these observed associations. Multiple comparisons were performed, which increased the risk of statistical type II error. Although the accelerometer is considered the gold standard method for assessing PA levels, it is not possible to measure the context or type of activity performed. However, it is worth mentioning that an increase in the volume of PA, regardless of intensity, should be encouraged since our findings suggest that even low-intensity activities are related to reduced arterial stiffness.

## CONCLUSION

In conclusion, our results indicate that men with peripheral artery disease who spent more time performing light physical activities experienced lower arterial stiffness. However, blood pressure and autonomic modulation parameters were not related to sedentary behavior or different intensities of physical activity in either men or women.
